# Spatial diffusion of the 2015–2016 Zika, dengue and chikungunya epidemics in Rio de Janeiro Municipality, Brazil

**DOI:** 10.1017/S0950268819001250

**Published:** 2019-07-09

**Authors:** A. P. R. Dalvi, J. U. Braga

**Affiliations:** 1 Escola Nacional de Saude Publica Sergio Arouca, Fundação Oswaldo Cruz, Rio de Janeiro, Brazil; 2 Instituto de Medicina Social, Universidade do Estado do Rio de Janeiro, Rio de Janeiro, Brazil

**Keywords:** Chikungunya virus, dengue virus, spatio temporal analysis, Zika virus

## Abstract

Different countries, especially Brazil, that have faced recurrent dengue epidemics for decades and chikungunya epidemics since 2014, have had to restructure their health services to combat a triple epidemic of arboviruses – Zika, dengue and Chikungunya – transmitted by the same vector, mainly *Aedes aegypti*, in 2015–2016. Several efforts have been made to better understand these three arboviruses. Spatial analysis plays an important role in the knowledge of disease dynamics. The knowledge of the patterns of spatial diffusion of these three arboviruses during an epidemic can contribute to the planning of surveillance actions and control of these diseases. This study aimed to identify the spatial diffusion processes of these viruses in the context of the triple epidemic in 2015–2016 in Rio de Janeiro, Brazil. Two study designs were used: cross-sectional and ecological. Sequential Kernel maps, nearest-neighbour ratios calculated cumulatively over time, Moran global autocorrelation correlograms, and local autocorrelation changes over time were used to identify spatial diffusion patterns. The results suggested an expansion diffusion pattern for the three arboviruses during 2015–2016 in Rio de Janeiro. These findings can be considered for more effective control measures and for new studies on the dynamics of these three arboviruses.

## Introduction

The emergence and reemergence of diseases have always been a concern of researchers, managers and the general population worldwide. Several factors are attributed to the spread of pathogens, including climatic, socio-environmental and human mobility factors [1, 2]. Among the diseases transmitted by vectors, Zika, dengue and chikungunya deserve attention. Although the three arboviruses may have an asymptomatic presentation [3], their severe forms pose a great challenge to public health. Zika virus (ZIKV) emerged as a major threat in the Americas in 2015, generating serious cases of microcephaly in newborns [4, 5] and Guillain–Barré [6]. Chikungunya virus (CHIKV) fever can trigger neurological and joint problems for months [7]. Finally, dengue virus (DENV) infection can present as a haemorrhagic form, which can lead to death [8].

ZIKV, CHIKV and DENV are transmitted by the same vectors, mainly *Aedes aegypti*, and, as a result, their global distributions often overlap [9].

In 2015 and 2016, several Brazilian municipalities were challenged by an epidemic caused by the ZIKV that alarmed health professionals. The discovery of the severe forms of ZIKV infection and its dispersion throughout the Americas led the Pan American Health Organization and World Health Organization (PAHO/WHO) to declare an emergency of international importance in 2015 [10]. Brazilian municipalities then endemic for the other arboviruses – DENV and CHIKV – faced the introduction of a new virus, ZIKV, transmitted by vectors or via sexual and vertical routes [11].

Several efforts have been made to improve the knowledge of ZIKV, especially in the context of the triple epidemic, and spatial analysis plays an important role in the understanding of the dynamics of diseases. Various techniques may be employed, such as those used in the investigation of outbreaks or epidemics [12]. Spatial diffusion is characterised as a dynamic process of movement of a phenomenon in space and time, which occurs when a disease is transmitted to a new region [13].

The spatial diffusion process can be classified as contagious, expansion, relocation or hierarchical. The expansion or contagious patterns are characterised by the onset in a given region and the spread to adjacent areas so that the disease has a greater intensity at the place of origin and spreads with less intensity to neighbouring areas. In contrast, hierarchical and relocation patterns are characterised by the onset of the disease in a certain place and a ‘jump’ of the disease to a more distant place. The process of diffusion may also be mixed, when both expansion and hierarchical diffusion processes are observed simultaneously [14]. Spatial diffusion provides information about the transmission dynamics of the disease, can support the planning of surveillance and control actions, and can be used to generate hypotheses for studies [15] and ultimately reducing the risk of disease spread [16].

Considering the occurrence and importance of the triple epidemic in Brazil, the objective of this study was to identify the spatial diffusion pattern of the diseases caused by ZIKV, DENV and CHIKV in 2015–2016 in Rio de Janeiro, Brazil.

## Methods

### Study design

This study used two approaches: (a) a cross-sectional study of the point spatial data of cases in the city of Rio de Janeiro, Brazil, and (b) an ecological study of the incidence rate of the population of the districts of Rio de Janeiro.

### Location, study period and data source

**Fig. 1.**
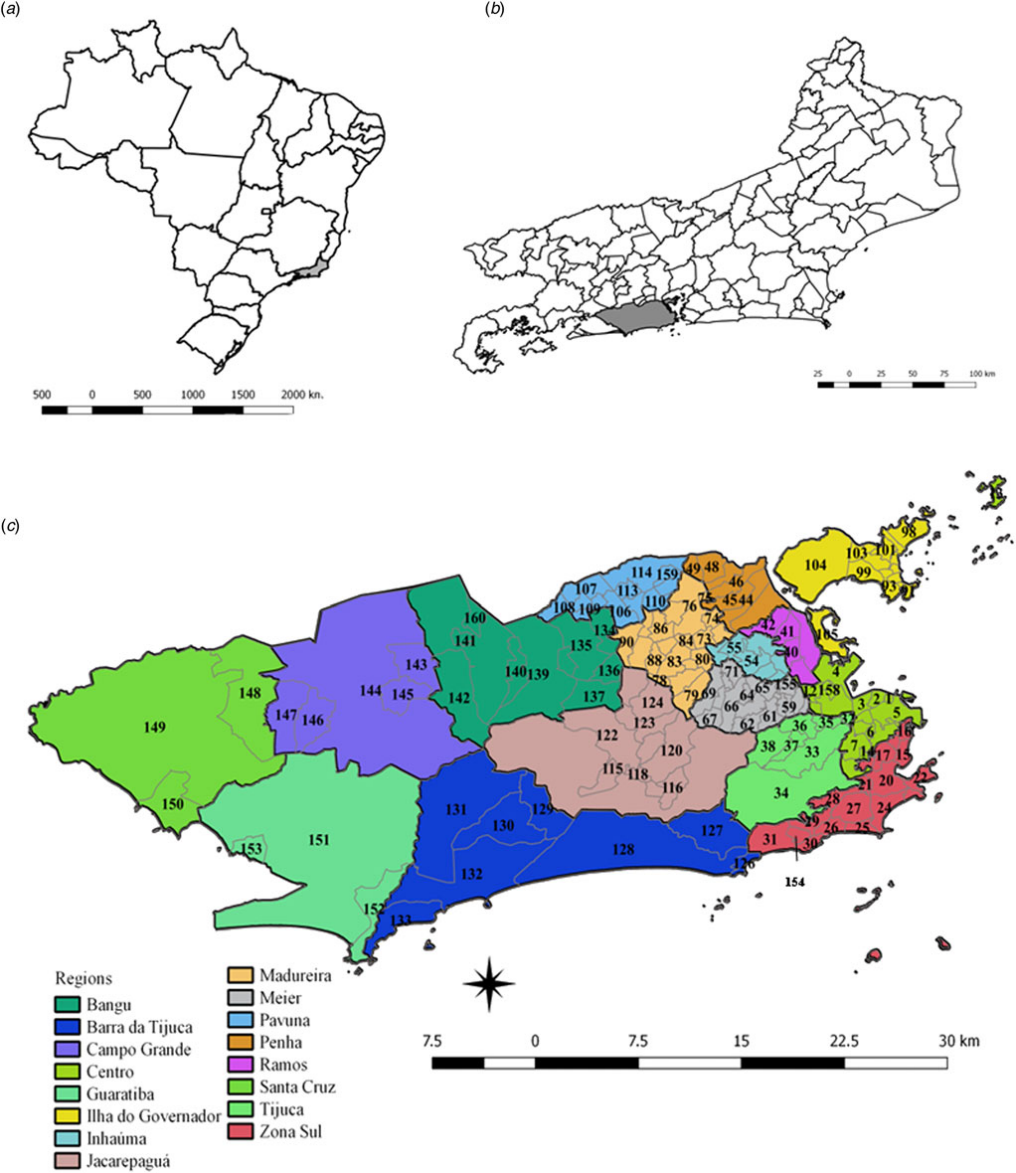
Maps of Brazil, and the State and Municipality of Rio de Janeiro, with divisions by planning regions and neighbourhoods. (a) Map of Brazil with divisions by State. The State of Rio de Janeiro is indicated in grey. (b) Map of the State of Rio de Janeiro with divisions by the municipality. The municipality of Rio de Janeiro is shown in grey. (c) Municipality of Rio de Janeiro with divisions by planning regions (colours) and by neighbourhoods (codes).


The patterns of spatial diffusion of the three arboviruses were studied in the municipality of Rio de Janeiro, Brazil, during the 2015–2016 epidemic. The municipality of Rio de Janeiro is located in the southeast region of the country (latitude 22°54′10″S and longitude 43°12′27″W) (Fig. 1). It has 1224.6 km^2^, with 48.6% of an urbanised area, and 31.4% of forest cover and 2.1% of water bodies in the non-urbanised area. The map showing the land cover municipality characteristics of urbanised and non-urbanised areas can be found in the link http://www.data.rio/datasets/mapa-de-uso-do-solo-do-município-do-rio-de-janeiro-2016 [17].

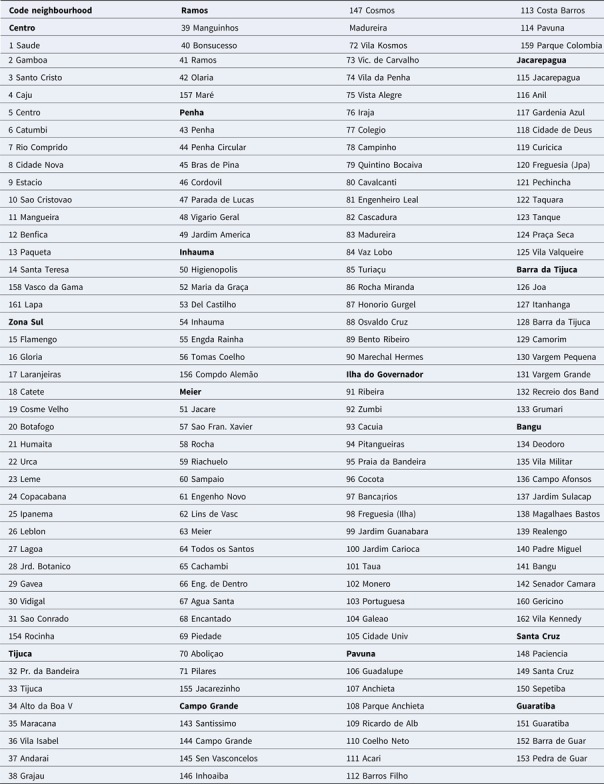


Rio de Janeiro is the second most populous city in Brazil, with a resident population of 6 320 446 and a density of 5265.82 inhabitants per km^2^, according to the 2010 census. It presents 94.4% of households with adequate sanitary sewage and 78.4% of urban households on public roads with adequate urbanisation (presence of manhole, sidewalk, paving and curb) [18]. The HDI-M (Human Development Index of the municipality) is 0.799 [18], being 0.604 the lowest and 0.959 the highest HDI-M within the municipality [19].

Zika, dengue and chikungunya cases notified to the national notifiable disease surveillance system (SINAN) of the Ministry of Health of Brazil in 2015 and 2016 were used. Individual records were obtained with authorisation. Cases confirmed by laboratory or clinical epidemiological criteria for each of the three arboviruses were included in the analysis.

The digital maps by districts of the municipality of Rio de Janeiro were extracted from the site of the municipality of Rio de Janeiro (http://www.data.rio/). The populations living in the neighbourhoods were estimated based on the 2010 census of the IBGE (Instituto Brasileiro de Geografia e Estatística) [18].

### Geocoding

The residential addresses of Zika, chikungunya and dengue cases were geocoded by Google Maps and Open Street Map (both by the QGIS Software MMQGIS plugin), Google Earth Pro, Bing and Batchgeo APIs. The coordinates resulting from Google Maps API classified as good quality (coordinate identification by street name and number) were initially included. Second, the coordinates obtained from the Open Street Map API that obtained the coordinates from the street name and number were used for observations with missing latitude and longitude data. Third, the addresses geocoded by the Google Earth Pro API were included when data were still missing; lastly, the Bing API was used for observations without coordinates. At the end of this process, all reports of ZIKV, DENV and CHIKV infections had coordinates. Then, an evaluation of the data that obtained duplicate coordinates was done. The observations that presented a large number of duplications (>8) were geocoded by Batchgeo and incorporated after correction.

### Spatial data analysis

The point pattern data were analysed by the sequential kernel maps [20] and nearest-neighbour analysis regression [21]. The area data were analysed by global autocorrelation correlogram using Moran's *I* autocorrelation [22] and local autocorrelation by analysing changes in the local indicators of spatial association (LISA) over time [23].

**Fig. 2.**
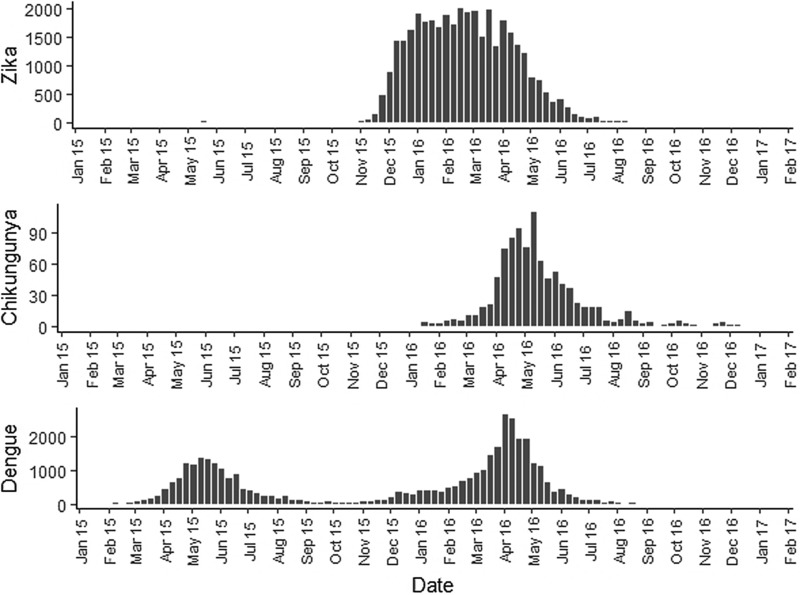
Zika, chikungunya and dengue epidemic curves for the Rio de Janeiro municipality in 2015 and 2016.

The sequential kernel maps were made and analysed for every four epidemiological weeks (EW) for the three arboviruses according to the evolution stages of the epidemic curves (Fig. 2). Analyses were performed for the following periods: EW 40 of 2015 to 39 of 2016, from EW 01 of 2015 to 40 of 2016 and from EW 44 of 2015 to 51 of 2016, for Zika, dengue and chikungunya, respectively. The two dengue epidemics occurring in the study period were analysed separately from EW 01 in 2015 to 40 in 2015 for the first epidemic curve and from EW 41 in 2015 to 40 in 2016 for the second epidemic wave. The kernel density estimator is a smoothing technique that uses point data and calculates the density of a given event per unit area, allowing the identification of hotspots (areas with a large number of events), on the map [20]. When sequential maps for several moments in time are made, it is possible to observe how the disease spreads over time in the study location. An exploratory analysis was performed for diffusion pattern detection. The maps were made using QGIS Software version 2.18 and the Heatmap plugin [24].

The second technique was proposed by Lee *et al*. [21], in which the cumulative nearest-neighbour index (NNI) variation is analysed when each point is added in time during the study period. In the analysis of the nearest-neighbour ratio, a comparison is made between the average distance observed between each point and its nearest neighbours and the expected average distance in a situation of spatial randomness [25]. Lee *et al*. proposed a technique in which the variation of the ratio of the nearest neighbour is first analysed when each point is added in time during the study period. The resulting curve of this variation is then adjusted to a regression curve that best fits its distribution. The distribution of the values in the graph is adjusted to a set of mathematical curves constructed to identify which best fit this distribution. The best fit was evaluated by *R*^2^, the determination coefficient, and infers on the spatial diffusion model. In simulations, inverse or the *S* curves better fit expansion diffusion patterns, while a cubic curve indicates a hierarchical diffusion pattern [21]. For the analysis of this statistic, the sp and SpatialEco packages of the R software were used.

The global autocorrelation correlograms were calculated and interpreted as described by Lam *et al.* [22]. The global Moran's *I* was used to represent the spatial autocorrelation of the incidence rates. The spatial autocorrelation refers to the association of a variable with its location. The Moran's *I* statistic varies from −1 to 1, where a statistic is positive when nearby areas have similar attributes, negative when assigned values are not similar and close to zero when the values are arranged randomly in space, indicating spatial independence [22]. The spatial neighbourhood condition was defined in this study by an adjacency matrix Wij. Spatial correlograms are diagrams showing spatial autocorrelation on the *Y*-axis and spatial scale, or lag, is computed on the *X*-axis. The spatial diffusion pattern is revealed in these diagrams. If the calculated correlograms show a decline as the lags move towards, the spatial diffusion pattern indicated is expansion. Curves, which show a decline and, after some lags, an increase, presenting a ‘*V*’ shape, indicate a hierarchical diffusion process [22]. The spdep package in R Software was used to perform this analysis.

Finally, the spatial diffusion pattern was also identified based on the analysis of LISA changes over time, as described by Cohen and Tita [23]. For this technique, the LISA was calculated for every four EW, similar to those used in the Kernel analyses. The local autocorrelation indicators for each observation indicate spatial clusters of similar values around an observation. In this way, the LISA will compare the incidence rate of a unit with the average rate of its neighbours and tests if this similarity is statistically significant. Statistical significance indicates spatial autocorrelation [26]. Cohen and Tita proposed in 1999 that the spatial diffusion patterns could be detected by observing LISA changes over time [23]. Initially the LISA values for each pair, composed of a given site and its neighbours, were evaluated according to the occurrence of local agglomeration and the occurrence of the pairs were identified according to the following indications: LOW-LOW (LL), a location with an attribute value below the average and neighbours with values below the average; LOW-HIGH (LH), a location with an attribute value below the average and neighbours with values above the average; HIGH-HIGH (HH), a location with an attribute value above the average and neighbours with values above the average; and HIGH-LOW (HL), a location with an attribute value above the average and neighbours with values below the average. This process was repeated for successive observations over time and then the changes in the levels of spatial association between each observation and its neighbours throughout the study period were verified. These changes indicate the pattern of spatial diffusion. GeoDa Software version 1.18.14 was used to perform this analysis [27].

### Ethical aspects

This project was developed in accordance with the guidelines of Resolution 466 of December 2012 and was submitted to the Ethics Committee of the National School of Public Health Sérgio Arouca of FIOCRUZ and authorised by the CAAE (n^o^ 85031718,2,0000,5240; Decision n^o^ 2,580,613 of 4 April 2018).

## Results

### Georeferencing

**Fig. 3.**
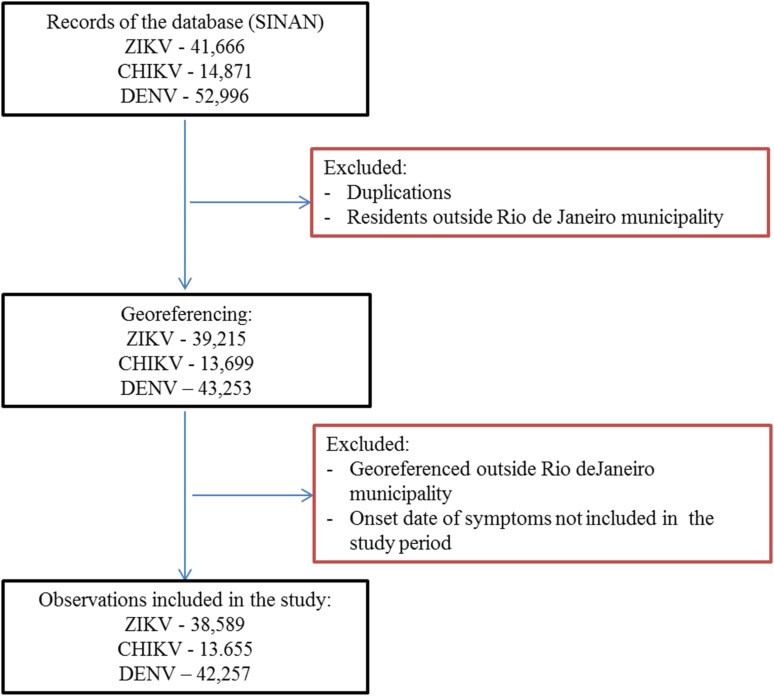
Georeferences of registered cases of ZIKV, CHIKV and DENV infections in Rio de Janeiro.

First, duplications and observations of individuals with notification of residence outside the municipality of Rio de Janeiro were removed. After georeferencing, localities outside of the municipality were identified and excluded. Among the georeferenced observations, 573 cases of Zika, 156 cases of chikungunya and 501 cases of dengue were reported mainly among residents of Niterói and Baixada Fluminense municipalities, which neighbour Rio de Janeiro city. Data with onset dates of symptoms outside the study period were also removed (Fig. 3). The proportion of cases georeferenced by API is shown in Table 1.

**Table 1.** Geocoding methods for Zika, chikungunya and dengue cases

Geocoding methods	Zika		Chikungunya		Dengue	
	*N*	%	*N*	%	*N*	%
Google Maps	8116	21.0	2276	16.7	3912	9.2
Open Street Map	26	0.1	10	0.1	119	0.3
Google Earth Pro	22 827	59.1	9239	67.6	30 769	72.8
Bing	7397	19.2	2130	15.6	7425	17.6
Batchgeo	223	0.6	–	–	32	0.1
TOTAL	38 589	100.0	13 655	100.0	42 257	100.0

Among ZIKV infections, 203 cases lacked a street name and were georeferenced by neighbourhood, while 1283 lacked household information and were georeferenced by street name.

Among CHIKV infections, 20 cases lacked a street name and were georeferenced by the neighbourhood, while 292 lacked household information and were georeferenced by street name. Chikungunya had the fewest duplications, was reported in the communities of Rio de Janeiro and had a poor return in the Batchgeo.

Among DENV infections, 190 cases lacked a street name and were georeferenced by the neighbourhood, 59 lacked contain street or neighbourhood name information and were georeferenced by the municipality, and 1074 lacked household information and were georeferenced only by street name.

### Point data analysis

**Fig. 4.**
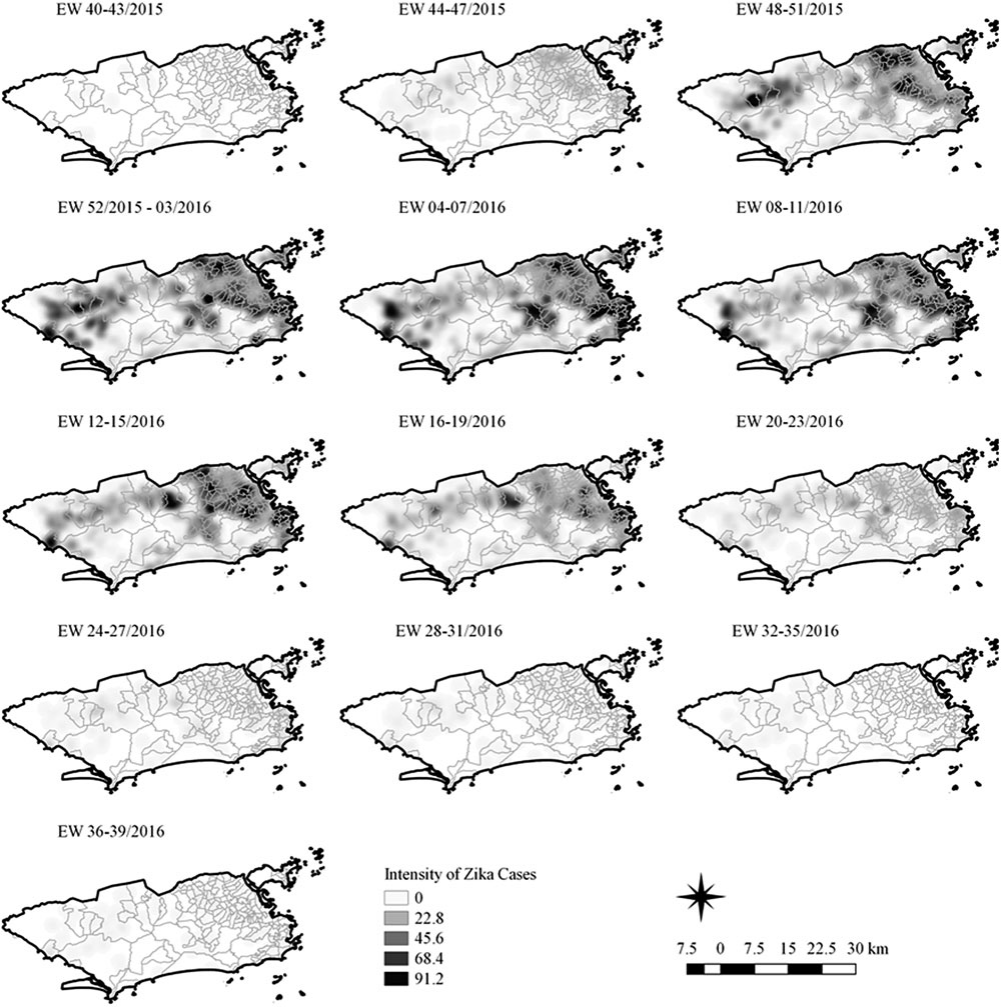
Sequential kernel maps of Zika cases from epidemiologic weeks 40 of 2015 to 39 of 2016.

**Fig. 5.**
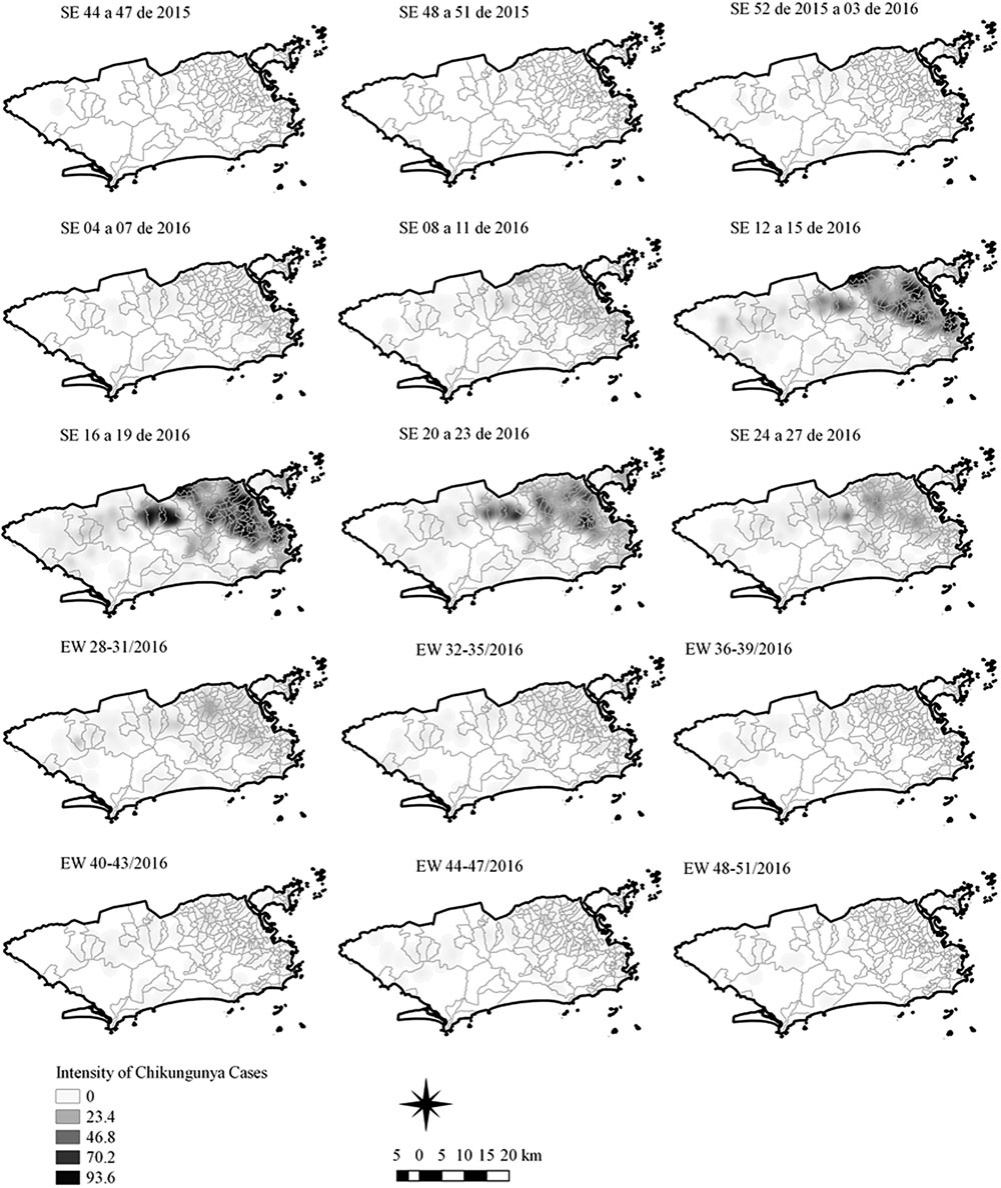
Sequential kernel maps of Chikungunya cases from epidemiologic weeks 44 of 2015 to 51 of 2016.

**Fig. 6.**
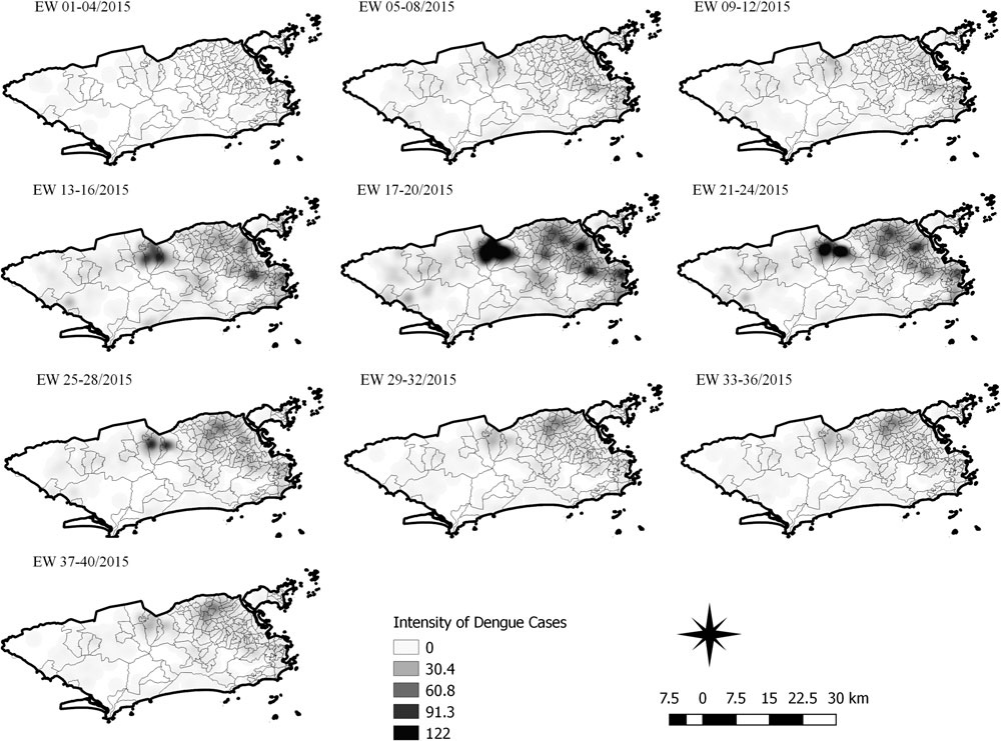
Sequential kernel maps for dengue for epidemiological weeks 01 of 2015 to 40 of 2015.

**Fig. 7.**
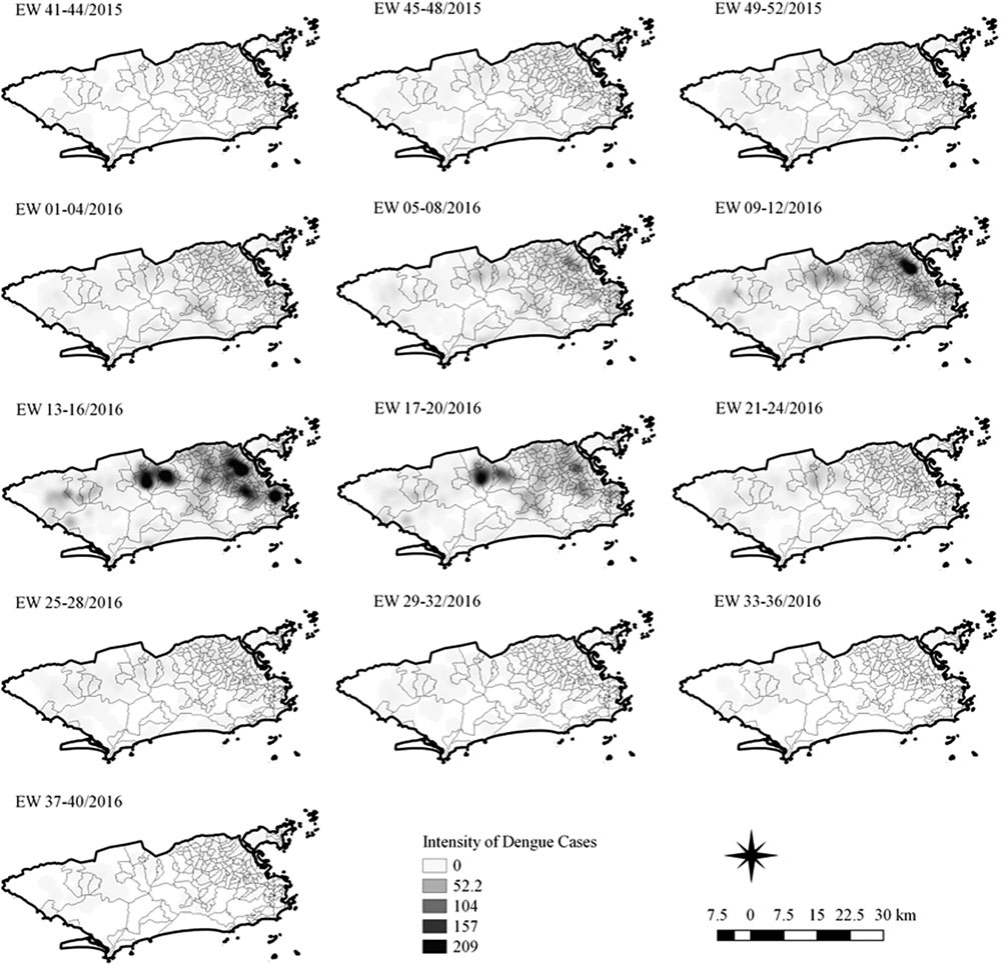
Sequential kernel maps for dengue for epidemiological weeks 41 of 2015 to 40 of 2016.

Sequential Kernel maps were made for every four EW for each disease under study and analysed visually according to the regions of the county (Figs 4–7).

Sequential maps of Zika showed a greater intensity of cases among the 48 EW 2015 to 19 EW in 2016. From EW 48 to 51 in 2015, hotspots were observed around the neighbourhoods of the planning regions of Pavuna, Penha and Madureira. In the following weeks, areas with greater intensity emerged in Sepetiba, Praça Seca, Rocinha and the region encompassing the planning region of Centro. EW 04 to 07 of 2016 comprised the peak of the epidemic curve, with the intensity remaining high in the regions of Sepetiba, Rocinha and in the area that encompasses the neighbourhoods of Flamengo, Catete, Santa Teresa, Rio Comprido, Estácio and Tijuca. Increased intensity was also observed in the regions of Penha, Santa Cruz districts, Copacabana and Botafogo. Through all study period, the hotspot of Zika cases not only remains in initial areas but also reached neighbouring areas, suggesting a putative expansion diffusion pattern.

The sequential maps of chikungunya showed an increased intensity of cases beginning in EW 04 to 07 of 2016 mainly in the area that included the neighbourhoods of the planning regions of Centro to Pavuna. Hotspots appeared in EW 12 to 15 EW in 2016 in the regions around Anchieta, Engenho Novo, Complexo do Alemão and Centro. The highest numbers of cases occurred in EW 16 to 19 in 2016, with hotspots in the same regions of the previous weeks and increased intensity in the regions of Bangu, Madureira and Penha. The propagation of cases restricted to certain areas suggested an expansion diffusion pattern.

The sequential maps for the first epidemic curve of dengue showed a higher intensity of cases from EW 13 to 16 of 2015, with moderate intensity in the region of Realengo and Vila Isabel. In EW 17 to 20 of 2015, the peak of the epidemic curve, hotspots occurred in the region encompassing the neighbourhoods of Bangu, Complexo do Alemão and Vila Isabel and, with less intensity, in the Centre region. Visual analysis of the sequential kernel maps referring to the first epidemic curve showed no disease jumps, suggesting a diffusion pattern of expansion.

In the second epidemic curve of dengue, low intensity of cases was observed in the Penha region from EW 05 to 08 of 2016. This intensity increased in the following weeks, from EW 09 to 12 of 2016, from Penha to Bonsucesso. In EW 13 to 16 of 2016, the peak of the epidemic curve, hotspots were observed in the regions of Realengo, Bangu, Vila Isabel, Engenho Novo, Centro regions and Penha to Inhaúma. In the following EW, the intensity of the cases decreased in almost all municipalities and was concentrated only in the Bangu and Senador Camará regions. Jumps of case intensity were not observed, suggesting a diffusion pattern of expansion.

**Fig. 8.**
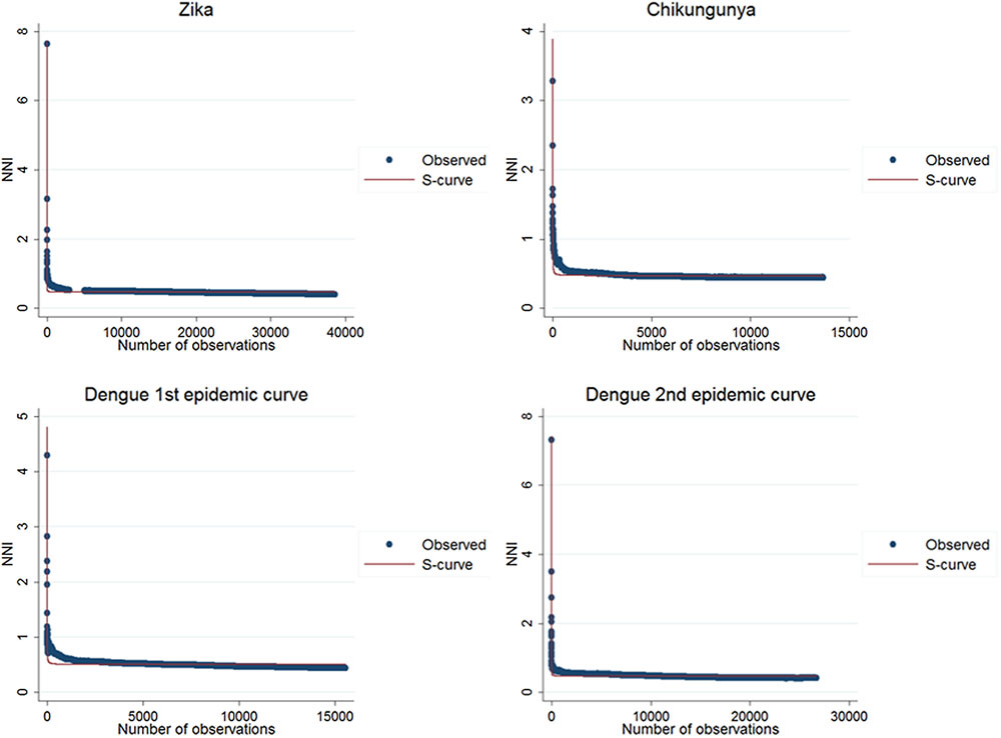
Best fit resulting curves generated by the nearest-neighbour variation over time for Zika, chikungunya and dengue.

The results of the nearest-neighbour regression analysis over time for Zika, chikungunya and the first and second dengue epidemic curves performed as described by Lee *et al*. indicated an expansion type of diffusion pattern for all three diseases. Among the curves that indicated a spatial diffusion pattern, the *S*-curve had the highest *R*^2^ value (Table 2, Fig. 8).

**Table 2.** Coefficients of determination of regression models for variation of the ratio of the nearest neighbour over time for Zika, chikungunya and dengue, Rio de Janeiro, 2015–2016

Disease	Inverse curve	Cubic curve	*S* curve
Zika	0.462*	0.567*	0.986*
Chikungunya	0.648*	0.569*	0.989*
Dengue 1st epidemic curve	0.478*	0.685*	0.984*
Dengue 2nd epidemic curve	0.550*	0.564*	0.984*

*P-value < 0.001

The results of LISA variations over time suggested a spatial diffusion pattern by the expansion of ZIKV and DENV infections in both CE and a hierarchical pattern for CHIKV (Table 3).

**Table 3.** Frequency of changes in LISA autocorrelation indexes for Zika, chikungunya and dengue incidence rates in Rio de Janeiro neighbourhoods, 2015–2016

Pattern	Zika		Chikungunya		Dengue 1st epidemic curve		Dengue 2nd epidemic curve	
	*N*	%	*N*	%	*N*	%	*N*	%
Expansion	11	61	11	34	13	57	10	56
Hierarchical	7	39	21	66	10	43	8	44
Total	18	100	32	100	23	100	18	100

**Fig. 9.**
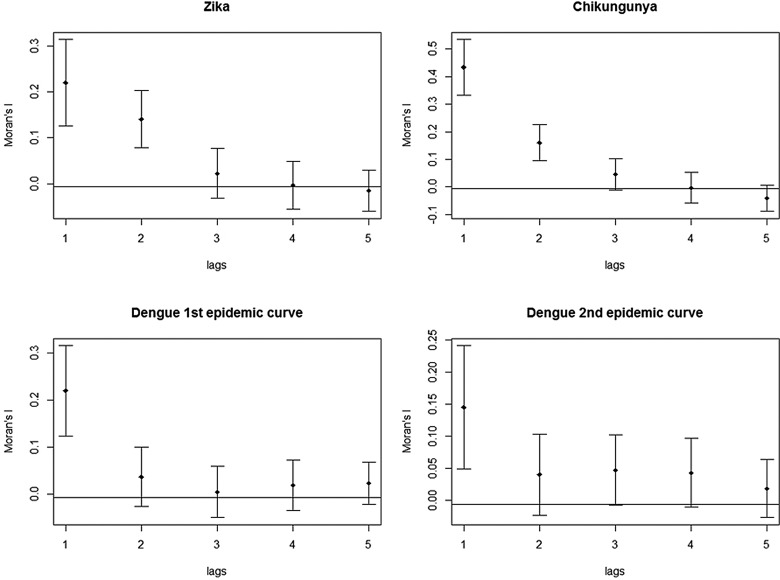
Correlograms of the global Moran's *I* for the incidence rates of Zika, chikungunya and dengue in Rio de Janeiro, 2015–2016.

The correlograms for Zika, chikungunya and dengue showed a decline in Moran's *I*, after the second spatial lag. The graph for Zika and chikungunya showed that this pattern persisted until the fifth spatial lag. In both plots of the two dengue epidemics, there was a stabilisation of Moran's *I* after the third spatial lag, still suggestive of an expansion diffusion pattern (Fig. 9).

These techniques showed that the spatial diffusion pattern of the three arboviruses during the 2015–2016 epidemics matched the expansion diffusion model.

## Discussion

The spatial diffusion pattern of expansion for Zika, chikungunya and dengue was suggested by the results of the Kernel sequential maps, the NNI variation over time, spatial autocorrelation correlogram and the LISA changes over time for the municipality of Rio de Janeiro in the 2015–2016 period.

The study period comprised two epidemic waves of dengue because both occurred during the Zika and chikungunya epidemics. This approach enabled the study of the dengue diffusion pattern in a scenario where there is an indication of problems in the classification of cases and to compare the two epidemics. Our results indicate that there was no change in the diffusion patterns in the two dengue waves studied and in the findings for Zika and chikungunya.

Several studies have reported problems related to the notification of Zika, chikungunya and dengue cases in Brazil. Although the Ministry of Health of Brazil only reported cases of Zika from April 2015 [28], ZIKV was identified in Brazil in 2013 [29, 30]. According to Brito *et al*., many Zika cases were reported as dengue in 2015 [31] and, as suggested by Teixeira *et al.*, many cases of chikungunya were reported as dengue in 2014 [32]. In addition, the Brazilian Ministry of Health indicated the underreporting of dengue cases [33]. The misclassification of cases was also described in a study in Gabon, Central Africa, where the presence of ZIKV was identified during dengue and chikungunya epidemics in 2007, prior to the initiation of notifications for ZIKV infections [34].

A review of the case definitions in use when the Zika epidemic arrived in Brazil concluded that they may have influenced the problems in the classification of cases during the study period since laboratory testing of all cases was not performed during the epidemic. In 2017, Braga *et al.* suggested that the Zika case definition used by the Brazilian Ministry of Health did not present good specificity and sensitivity [35].

The underreporting of cases may have occurred because some ZIKV, DENV and CHIKV infections are asymptomatic [3] and misclassification may be due to the similar initial clinical signs and symptoms of the three arboviruses [36].

The sequential kernel maps revealed that some regions were more affected by certain diseases than others. The regions close to the neighbourhoods of Sepetiba and Rocinha had high intensities of Zika cases and low intensities of the other two viruses. In contrast, the districts of Realengo, Padre Miguel and Bangu had only high intensities of chikungunya and dengue cases in a similar period of time, EW 12–20/2016. The northern region of the municipality is heavily affected by the three diseases and the area comprising the neighbourhood of Pavuna had the highest density of Zika cases, while the density of chikungunya was highest in the region near Anchieta, neighbouring Pavuna the density of dengue was highest in Penha. The epidemic curves and the sequential kernel maps show an increase of CHIKV and DENV cases when ZIKV cases begin to decline in EW 12 of 2016 (Figs 2 and 4–7). The analysis of sequential kernel maps suggested a spatial diffusion by expansion for the three arboviruses.

A factor common to neighbourhoods with high case intensities was a lower socio-economic level. A positive relationship between low socio-economic level and high risk of dengue was reported in the state of São Paulo [37] and Zika in the state of Bahia [38].

Sequential kernel maps are widely used in the studies of dengue in Brazil [39, 40, 41]. An expansion diffusion pattern was reported by Barreto *et al*. in 2008 and Melo *et al*. in 2010 and a mixed pattern was suggested by Morato *et al*. in 2015 [41]. Although kernel density maps are considered a visual analysis, this descriptive step is important for the initial observation of the distribution of diseases in space and time. Considering its high subjectivity in the identification of spatial diffusion patterns, more empirical methods were also used.

The analysis of NNI variation over time, spatial autocorrelation correlograms and changes of LISA over time suggested an expansion spatial diffusion pattern for the three arboviruses, except for the LISA variation as a function of time for CHIKV, which suggested a hierarchical pattern. Therefore, we indicate an expansion diffusion pattern for the three arboviruses in the context of the triple epidemic. This identification reveals the characteristics related to the disease dynamics. According to Cromley and McLafferty [14], the expansion of a disease reflects a localised human interaction between an individual and their neighbours. In this case, the vector population may also be connected. Similarly, the hierarchical process reflects the movement of individuals, how they interact and their social and transport connections [14]. Characteristics such as environmental and vector factors, network connections and proportions of susceptible and infected individuals should be further studied for a better understanding of our findings. The importance of interactions through network connections in the spatial diffusion process has been widely described [15, 42]. The region of the municipality of Rio de Janeiro is largely connected by highways and railways, favouring the expansion of the three arboviruses.

The characteristics of the sites of origin and destination of the transmission linked to the vector, population density and socio-economic level are related to the factors related to the local transmission and dispersion of ZIKV [43]. The main vector responsible for the transmission of the three arboviruses in Brazil, *A. aegypti*, can be coinfected by all three viruses and can transmit them simultaneously. Ruckert *et al*. observed that mosquitoes coinfected with ZIKV and CHIKV had a lower viral load of ZIKV compared to that of CHIKV [9]. This finding indicates the need for future studies to determine whether vector capacity may explain why certain regions are more affected by one virus than by another.

The present study aimed to identify the diffusion pattern of Zika, chikungunya and dengue in the context of the 2015–2016 triple epidemic in the municipality of Rio de Janeiro. We also aimed to assess a model to identify the characteristics of the disease dynamics, to identify patterns for the perspective of prediction of a model in order to assist in the emergence of new hypotheses and to contribute to the elaboration of more accurate prevention programmes [13]. This project was not designed to assess the relevant patient immunological, vector, environmental or climatic factors to explain the model of transmission of these diseases.

An important limitation of this work is related to the quality of the georeferenced addresses. Some addresses were georeferenced by only the street name, resulting in coordinates in the middle of a street. This occurred mainly in poor communities. In these locations, there is often a main address with subdivisions in alleys, houses and apartments. The APIs used and the individual search of these addresses could not capture this subdivision since it is not an official division in the municipality. Clusters were observed in the places where this occurred; thus, we concluded that there was no influence on the results of the analyses.

The existence of four serotypes of DENV was not considered in this study. Their differences related to symptomatology, patient immune response and consequent transmission potential may generate differences in the diffusion pattern suggested as a result of this work.

The correlograms are graphs that demonstrate the behaviour of spatial autocorrelations of the incidence rate (the variable used in this study) to the lag of a neighbourhood order. The neighbourhoods were defined by an adjacency matrix and the correlograms comprised five spatial lags, to the fifth-order neighbours. The analysis was performed assuming a greater number of spatial lags, with no change in the diffusion pattern. The results obtained using this method corroborated those reported in other studies.

The method based on LISA modifications returned a low proportion of frequencies with statistical significance and did not obtain a great difference in the frequencies to detect the spatial diffusion process for most diseases and should be interpreted with caution.

The analysis of the nearest neighbour with respect to time is considered the most formal analysis for the detection of spatial diffusion patterns. The findings in this study of spatial diffusion by expansion corroborate that reported for dengue in Taiwan [21].

In conclusion, we suggest an expansion diffusion pattern for the Zika, chikungunya and dengue epidemics in the context of the 2015–2016 triple epidemic in the city of Rio de Janeiro, Brazil, as well as the sites most affected by the diseases. This finding may help in the elaboration of a more effective control programme as well as the elaboration of new studies to fill gaps in the dynamics of the three arboviruses.
